# Fungi in hair roots of *Vaccinium* spp. (Ericaceae) growing on decomposing wood: colonization patterns, identity, and in vitro symbiotic potential

**DOI:** 10.1007/s00572-023-01101-z

**Published:** 2023-01-26

**Authors:** Martin Vohník, Martina Réblová

**Affiliations:** 1 Department of Mycorrhizal Symbioses, Institute of Botany, Czech Academy of Sciences, Lesní 322, Průhonice, 252 43 Czechia; 2 Department of Taxonomy, Institute of Botany, Czech Academy of Sciences, Zámek 1, Průhonice, 252 43 Czechia

**Keywords:** Agaricales, Ericoid mycorrhiza, *Hyaloscypha*, *Mycena*, Root-associated fungi, Saprobic fungi

## Abstract

Most of our knowledge on the ericoid mycorrhizal (ErM) symbiosis comes from temperate heathlands characterized by acidic peaty soils and many experiments with a few ascomycetous fungi. However, ericaceous plants thrive in many other ecosystems and in temperate coniferous forests, their seedlings often prosper on decomposing wood. While wood is typically exploited by basidiomycetous ectomycorrhizal (EcM) and saprobic fungi, the role of ErM fungi (ErMF) is much less clear. We explored the cultivable mycobiota of surface sterilized hair roots of *Vaccinium* spp. growing on decomposing wood in two coniferous forests in Mid-Norway (Scandinavia) and Northern Bohemia (Central Europe). Obtained isolates were identified using molecular tools and their symbiotic potential was tested in vitro. While the detected community lacked the archetypal ErMF *Hyaloscypha hepaticicola* and the incidence of dark septate endophytes and EcM fungi was negligible, it comprised other frequent asexual ascomycetous ErMF, namely *H. variabilis* and *Oidiodendron maius*, together with several isolates displaying affinities to sexual saprobic *H. daedaleae* and *H. fuckelii*. Ascomycete-suppressing media revealed representatives of the saprobic basidiomycetous genera *Coprinellus*, *Gymnopilus*, *Mycena* (Agaricales), and *Hypochnicium* (Polyporales). In the resyntheses, the tested basidiomycetes occasionally penetrated the rhizodermal cells of their hosts but never formed ericoid mycorrhizae and in many cases overgrew and killed the inoculated seedlings. In contrast, a representative of the *H. daedaleae*/*H. fuckelii*-related isolates repeatedly formed what morphologically appears as the ErM symbiosis and supported host's growth. In conclusion, while basidiomycetous saprobic fungi have a potential to colonize healthy-looking ericaceous hair roots, the mode(-s) of their functioning remain obscure. For the first time, a lineage in *Hyaloscypha* s. str. (corresponding to the former *Hymenoscyphus ericae* aggregate) where sexual saprobes are intermingled with root symbionts has been revealed, shedding new light on the ecology and evolution of these prominent ascomycetous ErMF.

## Introduction

Most of our knowledge on the ericoid mycorrhizal (ErM) symbiosis comes from temperate heathlands of the Northern Hemisphere that are characterized by acidic peaty soils high in recalcitrant phenolic compounds and low in available mineral nutrients and from many experiments with a few readily cultivable ascomycetous ErM fungi (ErMF), especially *Hyaloscypha hepaticicola* and *Oidiodendron maius* (Leake and Read 1991; Smith and Read 2008). It is widely accepted that under this scenario, these ErMF benefit their core Ericaceae hosts (= members of the early anther inversion clade of Ericaceae as defined in Kron et al. (2002), in the following text as ericaceous hosts, plants, etc.) mainly through improving nutrient uptake and alleviating substrate toxicity (Read and Kerley 1995; Read 1996; Perotto et al. 2002). However, ericaceous plants co-dominate vegetation in many other ecosystems (Kron and Luteyn 2005) and the spectrum of potential ErMF is much wider (Leopold 2016; Vohník 2020). In some ecosystems, ericaceous hair roots lack or are not dominated by the archetypal ErMF *H. hepaticicola* and *O. maius* and their place is taken by other mycobionts whose functioning is not fully understood (see Bruzone et al. (2015) and references therein). These often represent novel fungal lineages that occur in less explored locations (e.g., Midgley et al. 2016, 2018; Leopold et al. 2021; Vohník et al. 2022) and/or are difficult to grow in pure culture, e.g., members of Chaetothyriales (Allen et al. 2003; Lukešová et al. 2015; Toju et al. 2016; Baba and Hirose 2021), Sebacinales (Allen et al. 2003; Selosse et al. 2007; Vohník et al. 2016; Griffin and Kernaghan 2022), and *Kurtia argillacea* in Hymenochaetales (Kolařík and Vohník 2018). It has been suggested that some of them may confer adaptations distinct from those provisioned by the so far investigated ascomycetous ErMF, e.g., the ability to degrade recalcitrant aromatic substrates like lignin (Vohník et al. 2012a), the second most abundant biopolymer on Earth (Baucher et al. 1998).

Except confirmed and probable ErMF, ericaceous plants associate with a plethora of root mycobionts with unknown symbiotic status, including typical ectomycorrhizal (EcM) and saprobic basidiomycetes (e.g., Allen et al. 2003; Bougoure et al. 2007; Walker et al. 2011; Grelet et al. 2017). Under artificial conditions, these may form intracellular hyphal loops or pegs in the rhizodermal cells of ericaceous plants (e.g., Walker et al. 2011; Villarreal-Ruiz et al. 2012; Vohník et al. 2012a; Grelet et al. 2017) and even support the growth of the inoculated plants (Vohník et al. 2012b; Grelet et al. 2017), but the eco-physiological significance of such observations remains unclear. The mechanisms behind these positive effects are unknown and may include a release of nutrients to the host´s rhizosphere through autolysis of their mycelium (Duclos et al. 1983) and mineralization of organically bound nutrients (Vohník et al. 2012b; Grelet et al. 2017). In addition, ericaceous plants often associate with the so-called dark septate endophytes (DSE), a miscellaneous group of ascomycetous mycobionts with melanized hyphae that are ubiquitous in the roots of boreal and temperate plants and whose effects range from positive to negative (Newsham 2011; Mayerhofer et al. 2013). DSE may form intracellular hyphal coils in the rhizodermis of ericaceous roots (Massicotte et al. 2005; Vohník and Albrechtová 2011; Lukešová et al. 2015) and similarly to non-ericaceous hosts, their effects range from slightly positive to neutral to negative (Vohník et al. 2003, 2005; Lukešová et al. 2015).

**Fig. 1:**
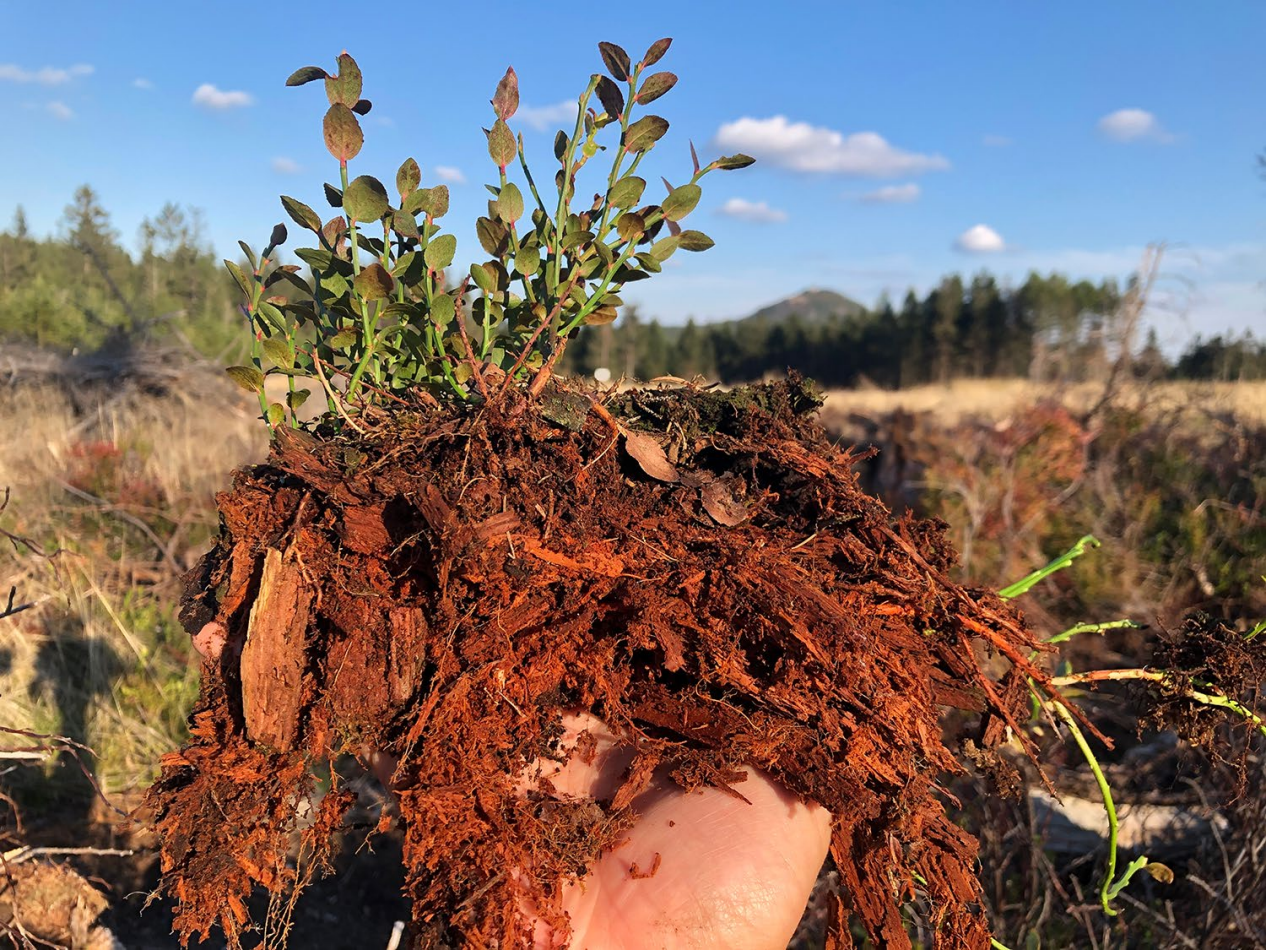
European blueberry (*Vaccinium myrtillus*) growing on decomposing wood, an overlooked substrate regularly colonized by many temperate ericaceous species. Next to nothing is known about the diversity of the fungi colonizing ericaceous hair roots growing in this substrate and the role these fungi play in wood decomposition. The picture was taken at the North Bohemian site investigated in this study (see Materials and methods), the peak in the background is Luž (Lausche in German, Łysa in Sorbian), the highest peak (793 m a. s. l.) of the Lusatian Mountains located at the border between Czechia and Germany

Ericaceous plants are not limited to acidic peaty substrates and in many European boreal and temperate forests, they often grow in soil mixtures comprising various volumes of decomposing wood (Fig. 1). Decaying tree stumps and thick branches laying on the forest floor seem to be especially suitable for the otherwise rare European blueberry (*Vaccinium myrtillus*) seedlings (Welch et al. 2000) as they retain moisture and provide elevated surfaces that assure an escape from competition with other forest floor plants (M. Vohník, personal observations). Dead wood forms a large part of the total biomass not only in boreal and temperate forests and represents an important pool of organically bound mineral nutrients (especially nitrogen and phosphorus) that are, however, directly non-accessible to primary producers and have to be released by heterotrophs, primarily basidiomycetous and to a lesser extent ascomycetous fungi (Boddy and Watkinson 1995). The major components of wood are cellulose, lignin and hemicelluloses and while many fungi are cellulolytic, they often cannot access lignin (Janusz et al. 2017; Goodell et al. 2020). Despite that the enzymatic repertoire of the so far investigated ascomycetous ErMF comprises enzymes involved in plant cell wall degradation (Cairney and Burke 1998; Perotto et al. 2018; Martino et al. 2018), their ability to degrade cellulose and especially lignin seems to be lower relative to the wood decomposing basidiomycetous saprobic fungi (Pearson and Read 1975; Bending and Read 1997). In contrast, *K. argillacea*, the basidiomycetous mycobiont forming sheathed ericoid mycorrhiza (a morphotype of ericoid mycorrhiza where intracellular hyphal coils in the host rhizodermis are accompanied by often multiple layers of thick hyphae with clamp connections on the root surface), has both cellulolytic and ligninolytic abilities (Vohník et al. 2012a). In addition, some Australian ErMF may outperform *H. hepaticicola* in utilization of phenolic compounds (Midgley et al. 2006).

To our knowledge, the root mycobiota of ericaceous plants growing on decomposing wood has not been investigated. Therefore, here we present the results of two surveys from Mid-Norway (Scandinavia) and Northern Bohemia (Central Europe) focused on fungi associated with *Vaccinium* spp. hair roots in this overlooked substrate. Both surveys were mainly observational, i.e., we did not rigorously test any specific hypotheses. On the other hand, thanks to the specific nature of decomposing wood, especially when compared to peat, we expected that the screened hair roots would harbor a spectrum of mycobionts differing from those commonly encountered in peatland ecosystems, possibly enriched in typical basidiomycetous EcM and saprobic fungi. The surveys were accompanied by microscopic observations of the root colonization and two in vitro resynthesis experiments and their results are presented together with the diversity and phylogenetic data.

## Materials and methods

### Sampling

A first set of samples was collected in May 2011 in a naturally regenerating Norway spruce (*Picea abies*) forest at the foothill of Forbordfjellet (N63.51877, E10.88811; ca. 400 m above sea level) close to Stjørdal in Mid-Norway. Mixed roots of European blueberry (*Vaccinium myrtillus*) and cowberry (*V. vitis-idaea*) overgrowing a partially decomposed spruce stump were collected and processed as described in Vohník et al. (2012a) with a special focus on evaluating the presence of sheathed ericoid mycorrhiza and isolating basidiomycetous root-associated fungi. A second set was collected in June 2011 in a secondary Norway spruce forest in Northern Bohemia, Czechia (N50.85593, E14.61735; ca. 664 m a. s. l.; in 2022, all adult spruce trees were cut down). Roots of 12 ca. 3-year-old European blueberry seedlings growing on decomposing thick branches in four slash piles left after a thinning were collected and processed in the same manner as above with a focus on describing their fungal colonization and isolating their ascomycetous and basidiomycetous mycobionts.

Herbarium specimens (as dried cultures) were deposited in the Herbarium of the Institute of Botany Institute, Czech Academy of Sciences in Průhonice (PRA), cultures were accessioned into Westerdijk Fungal Biodiversity Institute in Utrecht, the Netherlands (CBS).

### Microscopic observations of natural colonization

The root samples were washed under running tap water, ca. one half of both sets was cleared with 10% KOH for 15 min at 121 °C, briefly acidified with 3% HCl and washed under running tap water. The roots from Northern Bohemia were further stained with 0.05% trypan blue in lactoglycerol (glycerol + lactic acid + water, volume ratio 2:1:2) overnight and de-stained in lactoglycerol. Microscopic observations of the roots were performed with a compound Olympus BX60 microscope equipped with differential interference contrast at 400 × and 1000 × magnification. Photos were taken with an Olympus DP70 camera using QuickPHOTO MICRO v. 3.2 (Promicra) and the embedded Deep Focus Mode was employed when needed. The obtained photos were modified for clarity and contrast as needed and assembled into figures using Paint.net v. 4.0.13 (dotPDN LLC, Rick Brewster and contributors).

### Isolation of mycobionts

The remaining washed roots were surface sterilized for 30 s in 10% SAVO (common household bleach; Unilever; 100% SAVO contains 47 g/kg, i.e., 4.7%, sodium hypochlorite, NaClO), 3 × washed with sterile deionized water and ca. 2.5 mm segments of hair roots were plated on the surface of a growth medium [modified Melin Norkrans medium (MMN); Molina and Palmer (1982)] amended with Novobiocin sodium salt (50 mg/l; Sigma-Aldrich) to prevent growth of bacteria and incubated in the dark at room temperature for ca. two months. The segments from Northern Bohemia were cultivated on MMN with and without benomyl (4 mg/l, reduces growth of most ascomycetes; Sigma-Aldrich) whereas the segments from Mid-Norway were incubated only on MMN with benomyl. Sporulating mycelia were discarded and those non-sporulating were transferred to new dishes with MMN and used for molecular identification.

### DNA extraction, PCR amplification and sequencing

DNA was extracted from all isolates using an Extract-N-Amp Plant Kit (Sigma-Aldrich) following the manufacturer’s instructions. The ITS1-5.8S-ITS2 nuclear ribosomal DNA (rDNA) region was amplified using the ITS1F + ITS4 primer pair, with PCR parameters and gel electrophoresis as described in Vohník et al. (2012a). The PCR products were purified and sequenced in Macrogen Europe Laboratory (Macrogen) using the ITS1, ITS1F and ITS4 primers.

### Identification of mycobionts

The obtained sequences were screened in Finch TV v. 1.4.0 (Geospiza) and manually edited. Subsequently, the Czech and the Norwegian ones were separately aligned in Bioedit v. 7.1.8 (Hall 1999) and clustered at 99% similarity in TOPALi (Biomathematiscs & Statistics Scotland) (Tables 1 and 2). Representative sequences of each cluster were subjected to BLASTn searches (Zhang et al. 2000) in GenBank at NCBI (Sayers et al. 2019) as detailed in (Vohník 2020). Sequences of several Czech isolates displayed affinities to *Hyaloscypha* s. str. but did not seem to belong to any of the so far described and sequenced species. The amplified ITS rDNA of their representatives was sequenced with the ITS4 primer and LSU rDNA amplified with the LR0R + LR5 primer pair and sequenced using the same primers in Macrogen Europe Laboratory. Raw sequence data of these new hyaloscyphoid isolates were assembled and edited using Sequencher v. 5.4.6 (Gene Codes).

**Table 1:** Isolates obtained in this study from hair roots of *Vaccinium myrtillus* seedlings in Northern Bohemia

**OTU** **(number of isolates)**	**Representative isolate (ITS GenBank accession)**	Taxonomy*			**Probable lifestyle in ericaceous roots**	**Resyntheses**
NB-01 (19)	B78 (OP345138)	*Hyaloscypha variabilis*	Hyaloscyphaceae	Helotiales	ErM	R1
NB-02 (10)	B10 (OP345122)	*Hyaloscypha* sp.	Hyaloscyphaceae	Helotiales	ErM	R1 & R2
NB-03 (10)	B36 (OP345130)	*Pezicula* sp. (~ *P. neosporulosa*)	Dermateaceae	Helotiales	root-symbiotic/saprobic	—
NB-04 (6)	B56 (OP345133)	*Oidiodendron maius*	Myxotrichaceae	Ascomycota	ErM	R1
NB-05 (5)	B53 (OP345132)	*Tolypocladium album*	Ophiocordycipitaceae	Hypocreales	unknown	—
NB-06 (4)	B37 (OP345131)	*Metapochonia bulbillosa*	Clavicipitaceae	Hypocreales	root-symbiotic/unknown	—
NB-07 (2)	B12 (OP345123)	Leotiomycetes sp.	—	Leotiomycetes	unknown	—
NB-08 (1)	B27 (OP345128)	*Epicoccum* sp. (~ *E. nigrum*)	Didymellaceae	Pleosporales	root-symbiotic/unknown	—
NB-09 (1)	B73 (OP345137)	*Coprinellus* sp. (~ *C. disseminatus*)	Psathyrellaceae	Agaricales	root-symbiotic/saprobic	—
NB-10 (1)	B83 (OP345141)	*Gymnopilus* sp. (~ *G. penetrans*)	Strophariaceae	Agaricales	root-symbiotic/saprobic	R1 & R2
NB-11 (1)	B14 (OP345124)	*Mycena* sp. (~ *M. galopus*)	Mycenaceae	Agaricales	root-symbiotic/saprobic	R1 & R2
NB-12 (1)	B35 (OP345129)	*Cordana pauciseptata*	Cordanaceae	Cordanales	unknown	—
NB-13 (1)	B81 (OP345140)	*Diaporthe* sp.	Diaporthaceae	Diaporthales	unknown	—
NB-14 (1)	B25 (OP345127)	Pezizomycotina sp.	—	Pezizomycotina	unknown	—
NB-15 (1)	B20 (OP345125)	*Trichoderma* sp.	Hypocreaceae	Hypocreales	unknown	—
NB-16 (1)	B32 (NS)	PAC	Mollisiaceae	Helotiales	endophytic (DSE)	—

*DSE* dark septate endophyte, *ErM* ericoid mycorrhizal (based on morphological observations from in vitro experiments), *ITS* ITS1-5.8S-ITS2 rDNA, *NS* not submitted, *PAC Phialocephala fortinii* s. l. – *Acephala applanata* species complex, *R1* first resynthesis, *R2* second resynthesis

^*^According to MycoBank (mycobank.org), accessed 27/7/2022

**Table 2:** Isolates obtained in this study from hair roots of *Vaccinium* spp. in Mid-Norway

**OTU** **(number of isolates)**	**representative isolate (ITS GenBank accession)**	Taxonomy*			**Probable lifestyle in ericaceous roots**	**Resyntheses**
MN-01 (17)	JPK-117 (OP345143)	*Mycena* sp. (~ *M. galopus*)	Mycenaceae	Agaricales	root-symbiotic/saprobic	R2
MN-02 (3)	JPK-152 (OP345145)	*Mycena* sp. (~ *M. sanguinolenta*)	Mycenaceae	Agaricales	root-symbiotic/saprobic	R2
MN-03 (2)	JPK-123 (OP345144)	*Mycena* sp.	Mycenaceae	Agaricales	root-symbiotic/saprobic	—
MN-04 (1)	JPK-151 (OP863026)	*Mycena* sp. (~ *M. epipterygia*)	Mycenaceae	Agaricales	root-symbiotic/saprobic	R2
MN-05 (1)	JPK-155 (OP345147)	*Gymnopilus* sp. (~ *G. penetrans*)	Strophariaceae	Agaricales	root-symbiotic/saprobic	R2
MN-06 (1)	JPK-154 (OP345146)	*Gymnopilus* sp. (~ *G. penetrans*)	Strophariaceae	Agaricales	root-symbiotic/saprobic	—
MN-07 (1)	JPK-111 (OP345142)	*Hypochnicium* sp. (~ *H. geogenium*)	Meruliaceae	Polyporales	root-symbiotic/saprobic	—
MN-08 (1)	JPK-132 (OP863025)	*Serendipita* sp.	Serendipitaceae	Sebacinales	ErM	(Vohník et al. 2016)

*ErM* ericoid mycorrhizal (based on morphological observations from in vitro experiments), *ITS* ITS1-5.8S-ITS2 rDNA, *LSU* 28S rDNA, *R2* second resynthesis

^*^According to MycoBank (mycobank.org), accessed 27/7/2022

### Phylogenetic analyses

Since the new hyaloscyphoid isolates were sterile in culture and no diagnostic features were detected, their closest relatives were selected from the top ranked hits using BLASTn searches of the ITS and LSU sequences generated in this study. The homologous ITS and LSU sequences of representatives of Hyaloscyphaceae were retrieved from GenBank at NCBI. The GenBank accession numbers of all strains analyzed are listed in Table 3.

**Table 3:** Taxa, isolate information and new sequences determined for this study and additional sequences of members of Hyaloscyphaceae retrieved from GenBank for the phylogenetic analysis

**Taxon**	**Source**	**ITS**	**28S**	**Reference**
*Hyaloscypha alniseda*	CBS 123.91	MH018930	**OP340027**	Fehrer et al. (2019), this study
*Hyaloscypha bicolor*	CBS 116122 T	AJ430147	MH018942	Vrålstad et al. (2002), Fehrer et al. (2019)
*H. bicolor*	CBS 144009	MH018932	MH018943	Fehrer et al. (2019)
*Hyaloscypha* cf. *bulbopilosa*	KUS-F52573	JN033423	JN086726	Han et al. (2014)
*H.* cf. *bulbopilosa*	TNS-F18073	JN033451	JN086751	Han et al. (2014)
*Hyaloscypha daedaleae*	CBS 120.91	MH018927	**OP340026**	Fehrer et al. (2019), this study
*H. daedaleae*	CBS 121.91	MH018928	**OP340025**	Fehrer et al. (2019), this study
*H. daedaleae*	ZW-Geo138-Clark	AY789416	AY789415	Wang et al. (2005)
*Hyaloscypha epiporia*	CBS 125.91	MH018929	**OP340028**	Fehrer et al. (2019), this study
*Hyaloscypha finlandica*	ARON 2948.S	AJ292202	—	Vrålstad et al. (2000, 2002)
*H. finlandica*	CBS 444.86 IT	AF486119	MH018941	Grünig et al. (2008), Fehrer et al. (2019)
*Hyaloscypha fuckelii*	CBS 126292 (as M233)	EU940230	EU940154	Baral et al. (2009)
*H. fuckelii*	AMFB1780	MT231691	MT231691	Kosonen et al. (2021)
*H. fuckelii*	TK7053	MT231692	MT231692	Kosonen et al. (2021)
*Hyaloscypha gabretae*	CBS 145341 T	MZ520780	MZ520769	Vohník et al. (2022)
*H. gabretae*	CBS 146193	MZ520781	MZ520770	Vohník et al. (2022)
*H. gabretae*	CBS 146194	MZ520782	MZ520771	Vohník et al. (2022)
*Hyaloscypha gryndleri*	CBS 145336	MZ520784	MZ520773	Vohník et al. (2022)
*H. gryndleri*	CBS 145337 T	MZ520785	MZ520774	Vohník et al. (2022)
*H. gryndleri*	CBS 146192	MZ520790	MZ520779	Vohník et al. (2022)
*Hyaloscypha hepaticicola*	CBS 126283 (as M171)	EU940194	EU940118	Baral et al. (2009)
*H. hepaticicola*	CBS 126291 (as M339)	EU940226	EU940150	Baral et al. (2009)
*H. hepaticicola*	UAMH 6735* T	AY762620	MH018947	Hambleton and Sigler (2005), Fehrer et al. (2019)
*Hyaloscypha herbarum*	CBS 126.91	MH018931	**OP340030**	Fehrer et al. (2019), this study
*Hyaloscypha intacta*	TK7111	MT231694	MT231694	Kosonen et al. (2021)
*Hyaloscypha leuconica*	TK7014	MT231695	MT231695	Kosonen et al. (2021)
*Hyaloscypha melinii*	CBS 143705 T	EF093175	MH018946	Vohník et al. (2013), Fehrer et al. (2019)
*H. melinii*	SM7-1	EF093174	—	Vohník et al. (2013)
*Hyaloscypha monodictys*	TNS-F5013	JN033456	JN086756	Han et al. (2014)
*Hyaloscypha occulta*	TNS-F31287	JN033454	JN086754	Han et al. (2014)
*Hyaloscypha spiralis*	KUS-F52652	JN033426	JN086729	Han et al. (2014)
*H. spiralis*	TNS-F17909	JN033440	JN086741	Han et al. (2014)
*Hyaloscypha variabilis*	UAMH 8861 T	AY762619	MH018944	Hambleton and Sigler (2005), Fehrer et al. (2019)
*Hyaloscypha vitreola*	CBS 126275 (as M236)	EU940232	EU940156	Stenroos et al. (2010)
*H. vitreola*	CBS 126276 (as M39)	EU940231	EU940155	Stenroos et al. (2010)
*H. vitreola*	CBS 127.91	JN033378	JN086681	Han et al. (2014)
*H. vitreola*	M220	FJ477059	FJ477058	Baral et al. (2009)
*Hyaloscypha vraolstadiae*	UAMH 10111 T	AJ292199	MH018945	Vrålstad et al. (2002), Fehrer et al. (2019)
*H. vraolstadiae*	UAMH 11203	MH018933	**OP340029**	Fehrer et al. (2019), this study
*Hyaloscypha usitata*	TK7083	MT231696	MT231696	Kosonen et al. (2021)
*Hyaloscypha* sp.	B3	**OP345121**	—	this study
*Hyaloscypha* sp.	B10 (CBS 149189, PRA 21325)	**OP345122**	**OP340024**	this study
*Hyaloscypha* sp.	B23	**OP345126**	—	this study
*Hyaloscypha* sp.	B58	**OP345134**	—	this study
*Hyaloscypha* sp.	B62	**OP345135**	—	this study
*Hyaloscypha* sp.	B67	**OP345136**	—	this study
*Hyaloscypha* sp.	B80	**OP345139**	—	this study
*Hyphodiscus brachyconius*	CBS 700.73 T	GU727557	GU727557	Bogale et al. (2010)
*Hyphodiscus brevicollaris*	CBS 126.74 T	GU727561	GU727561	Bogale et al. (2010)
*Hyphodiscus luxurians*	CBS 647.75 T	GU727560	GU727560	Bogale et al. (2010)
Fungal sp.	2.20.4G	KM068412	—	Sarjala et al. (unpublished)
Fungal sp.	3.44.4 J	KJ649999	—	Sarjala et al. (unpublished)

T and IT after strain number indicate ex-type and ex-isotype strains, new sequences determined for this study are in bold

*CBS* Westerdijk Fungal Biodiversity Institute (formerly Centraalbureau voor Schimmelcultures), Utrecht, the Netherlands, *PRA* Herbarium of the Institute of Botany, Czech Academy of Sciences, Průhonice, Czechia

^*^Ex-type strain of *Pezizella ericae*

The ITS and LSU sequences were aligned in Mafft v. 7.487 (Katoh and Standley 2013) implemented in the CIPRES Science Gateway v. 3.3 (Miller et al. 2010) and manually corrected in Bioedit v. 7.1.8 (Hall 1999) when necessary. Single-locus ITS and LSU data sets for representatives of Hyaloscyphaceae (ITS: 52 sequences/536 characters including gaps, LSU: 41/1302), for which we assumed rate heterogeneity, were evaluated using MrModeltest v. 2.4 (Nylander 2004) to find the best partitioning scheme and to select best-fit models under the corrected Akaike information criteria. The SYM + G best-fit model was selected for both partitions. The concatenated ITS-LSU dataset (deposited in TreeBase 29,685) was subjected to phylogenetic analyses. The first 89 nucleotides of LSU at the 5′-end were excluded from the alignment because of the incompleteness in most sequences. The dataset consisted of 1838 characters including gaps and 343 unique character sites. Three members of the genus *Hyphodiscus* (*Hy*.) (Hyphodiscaceae, Helotiales), namely *Hy. brachyconius*, *Hy. brevicollaris* and *Hy. luxurians*, were used to root the tree.

Phylogenetic relationships were evaluated using maximum likelihood (ML) and Bayesian Inference (BI) analyses and were performed through the CIPRES Science Gateway v. 3.3. ML analysis was performed with RAxML-HPC v. 8.2.12 (Stamatakis 2014) with a GTRCAT approximation. Nodal support was determined by non-parametric bootstrapping (BS) with 1000 replicates. BI analysis was performed in a likelihood framework as implemented in MrBayes v. 3.2.6 (Huelsenbeck and Ronquist 2001) using default parameters. The B-MCMCMC analysis lasted until the average standard deviation of split frequencies was below 0.01 with trees saved every 1000 generations. The first 25% of saved trees, representing the burn-in phase of the analysis, were discarded. The remaining trees were used for calculating posterior probabilities (PP) of recovered branches. Obtained trees were viewed in SeaView v. 4 (Gouy et al. 2010) and edited in MS PowerPoint (Microsoft).

### First resynthesis

Mycelial cultures of five fungal isolates from Northern Bohemia (three ascomycetes and two basidiomycetes, Table 1) were pre-cultivated in Petri dishes on potato carrot agar (PCA) in the dark at room temperature for six weeks. Seeds of European blueberry of local origin were extracted from fresh fruits, surface sterilized 1 min in 10% SAVO, 3 × washed with sterile deionized water and left to germinate and develop on MMN with no maltose, 1 g/L glucose and 50 μg/L Novobiocin added to suppress possible bacterial growth in a growth chamber under a 21 °C – 16 h light/15 °C – 8 h dark cycle and irradiation of 200 μmol/m^2^ s^1^ for 3 months. The cultivation substrate consisted of peat, perlite, and a mixture of dead wood of local origin (extracted from an old Norway spruce stump in the Průhonice Park) and wood shavings (volume ratio 4:2:1). The substrate was added to Magenta GA-7 cultivation vessels (Sigma-Aldrich), 25 g per each vessel, and watered with 20 mL of sterile deionized water. The vessels with the substrate were double autoclaved (60 min at 121 °C repeated after 36 h) and when cooled down, plugs (diam. ca. 5 mm) excised from the mycelial cultures were added on the surface of the substrate (four plugs per each vessel). A control treatment was established with PCA plugs without fungal mycelium. Each vessel was watered with 10 mL of sterile deionized water, the plugs were mixed into the substrate and one blueberry seedling was inserted into the substrate per each vessel. There were five vessels per treatment, incl. the fungus-free control. The vessels were incubated in the same growth chamber under the same regime as above and periodically checked. The experiment was harvested after 137 days, the seedlings were gently removed from the substrate and their shoots were separated from the roots. The shoots were dried at 65 °C overnight and weighed. The roots were gently washed under running tap water to remove residues of the cultivation substrate and treated as described above for the naturally colonized roots, including microscopy. Three small pieces of the cultivation substrate were extracted from each vessel and placed on the surface of solidified malt extract agar (MEA, HiMedia) in plastic Petri dishes (9 cm in diam.) and incubated in the dark for 1 month. DNA was extracted from representative cultures and ITS rDNA was amplified and sequenced as described above. The obtained sequences were identified as described above and compared with the sequences of the inoculated fungi.

### Second resynthesis

Mycelial cultures of seven fungal isolates, three from Northern Bohemia and four from Mid-Norway (Tables 1 and 2), one ascomycete and six basidiomycetes, were pre-cultivated as above. The cultivation substrate consisted of peat and perlite (volume ratio 1:1) and 14 g of the substrate were added to each Magenta GA-7 vessel. The vessels were double autoclaved as above except that before the second autoclaving, 50 mL of molten 0.8% water agar amended with 0.1% active charcoal were pipetted over the substrate in each vessel. After cooling down, a small piece of substrate was extracted from 10 random vessels and the pieces were aseptically transferred to plastic Petri dishes (5 cm in diam.) with MEA. The vessels and the dishes were incubated for 2 weeks at room temperature in the dark to doublecheck their sterility and no fungal mycelium was detected upon final inspection. Subsequently, plugs (diam. ca. 3 mm) excised from the mycelial cultures were added on the surface of the substrate (nine plugs per each vessel), a control treatment obtaining PCA plugs without fungal mycelium. The vessels with the plugs were incubated as above and after two months, all plugs extracted from fungal cultures were covered with mycelium spreading over the surface of the cultivation substrate (control plugs did not produce any mycelium). The plugs were mixed into the substrate and one 3-months-old European blueberry seedling obtained as above was inserted into the substrate per each vessel. There were five vessels per treatment, incl. the fungus-free control. The vessels were incubated in the same growth chamber under the same regime as above and periodically checked. The experiment was harvested after 158 days and the seedlings were treated as described above.

### Statistical analyses

Because the datasets from the two resynthesis experiments violated the assumptions of ANOVA, the effects of inoculation on dry shoot weight (both resyntheses) and fresh root weight (second resynthesis) were evaluated using the non-parametric Kruskal–Wallis test followed by multiple comparisons of mean ranks (Dunn´s test) in STATISTICA v. 12 (Statsoft).

## Results

### Microscopic observations of natural colonization

**Fig. 2:**
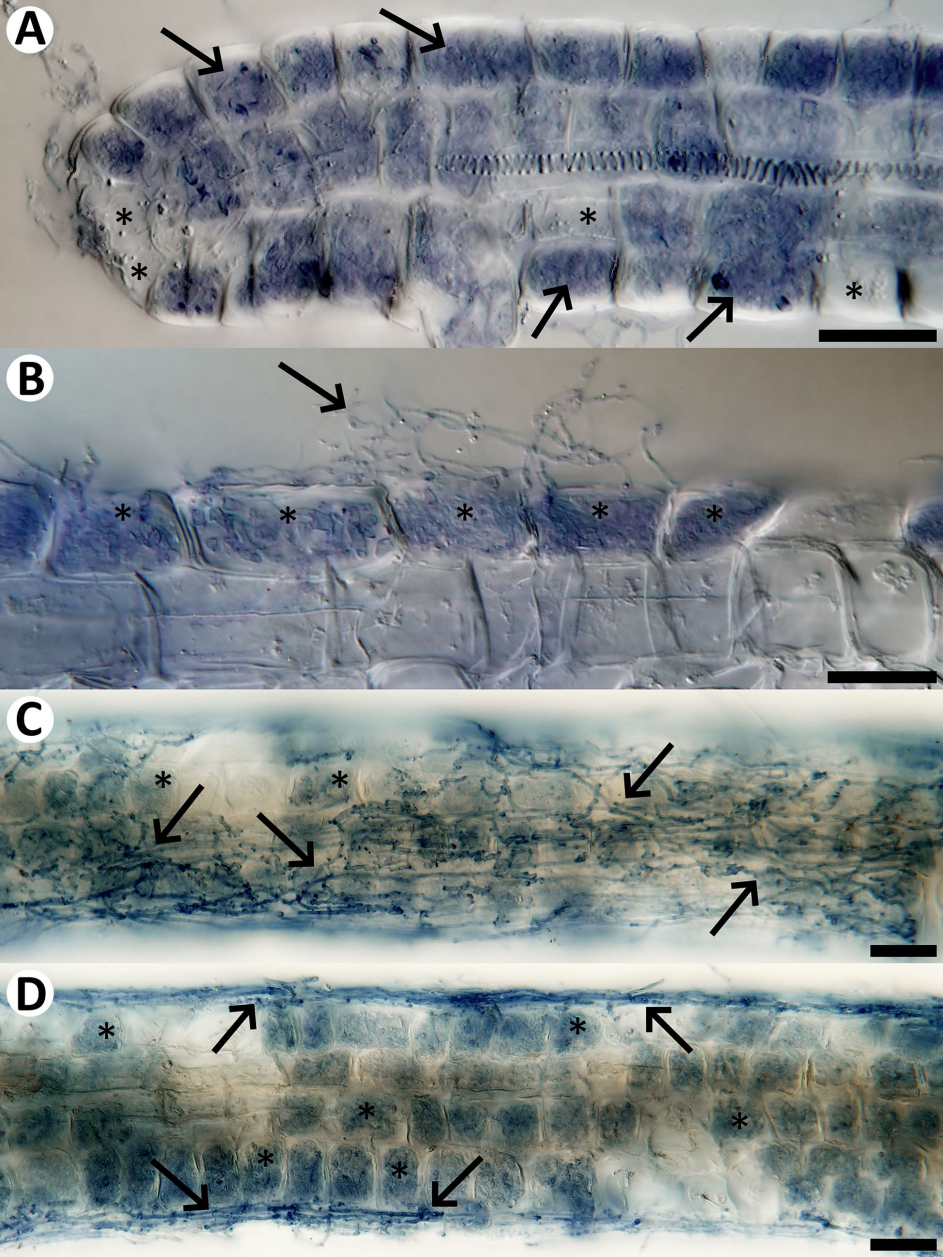
Natural fungal colonization in hair roots of *Vaccinium myrtillus* from Northern Bohemia.** A:** Typical ericoid mycorrhizal pattern, i.e., intracellular hyphal coils, present in most of the hair root´s rhizodermal cells (arrows point at some examples). Only a few rhizodermal cells remain without fungal colonization (examples marked with asterisks). **B:** Extraradical mycelium (arrow) accompanying ericoid mycorrhizal colonization (examples marked with asterisks). **C, D:** Sparse mantles formed by thin, often undulated hyphae on the surface of the hair root (arrows). Ericoid mycorrhizal colonization occurs just below the nets, in the host´s rhizodermis (examples marked with asterisks). All roots were cleared with 10% KOH, stained with trypan blue and observed using a compound microscope equipped with differential interference contrast as described in Materials and Methods. Scale bars = 20 μm

**Fig. 3:**
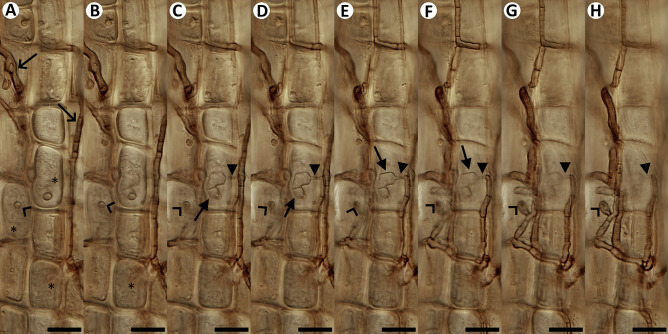
Natural fungal colonization in hair roots of *Vaccinium* spp. from Mid-Norway. Eight consecutive views of a hair root with dark septate hyphae on the surface (open arrows point at some examples in **A**) and intracellular hyphal colonization morphologically corresponding to ericoid mycorrhiza (examples in **A** and **B** marked with asterisks). **C**–**F**: An intracellular haustorium-like structure (closed arrows) is connected with a dark septate hypha on the surface through a thin penetration hypha (closed arrowheads in **C**–**H**). Note that the same rhizodermal cell is also occupied with dense hyphal coils (**A**, **B**). **A**–**H:** Another dark septate hypha on the surface is connected through a penetration hypha (open arrowheads) with intracellular hyphal colonization corresponding to ericoid mycorrhiza. The root was cleared with 10% KOH and observed using a compound microscope equipped with differential interference contrast as described in Materials and Methods. Scale bars = 20 μm

Sheathed ericoid mycorrhizae were found only in the samples from Mid-Norway. In both sets of samples, the hair roots displayed typical ericoid mycorrhizal colonization, i.e., dense intracellular hyphal coils in the rhizodermis. The colonization often started already at the tips of the hair roots (Fig. 2A) and in the samples from Northern Bohemia, it was often accompanied by extensive extraradical mycelium (Fig. 2B). In addition, many hair roots from Northern Bohemia were covered by sparse mantles formed by thin, often undulated hyphae that were connected with the dense intracellular hyphal coils in the rhizodermis (Fig. 2C and D). Both sets of samples contained thick, often interwoven hyphae with clamp connections, but these were never seen penetrating the screened roots (now shown). Unexpectedly, in both sets of samples there were no melanized microsclerotia typical of the dark septate endophytes. While the Czech samples also lacked thick melanized surface hyphae, these were regularly present in the Norwegian samples and in many cases, they seemed to be directly connected with much thinner intracellular hyphae occurring in the host´s rhizodermis and morphologically resembling ericoid mycorrhizae (Fig. 3). Sometimes, such connections seemed to be realized through haustoria-like intracellular structures (Fig. 3C‒G) that are, to our best knowledge, not known from resynthesis experiments with typical ErMF.

### Isolation and identification of mycobionts

The Czech samples yielded 65 isolates with high-quality sequences that were grouped into 16 OTU at 99% sequence similarity. The most abundant was *Hyaloscypha variabilis* (19 isolates) followed by *Hyaloscypha* sp. (10), *Pezicula* sp. (10), *Oidiodendron maius* (6), etc. Nine OTU were represented only by one isolate (Table 1). The Norwegian samples yielded 27 isolates grouped into 8 OTU and the most abundant was *Mycena* sp. (17). The three following OTU belonged to the same genus and in total comprised 6 isolates. Two OTU with a single isolate belonged to the genus *Gymnopilus*, one to *Hypochnicium* and one to *Serendipita* (Table 2).

### Phylogenetic analyses

**Fig. 4:**
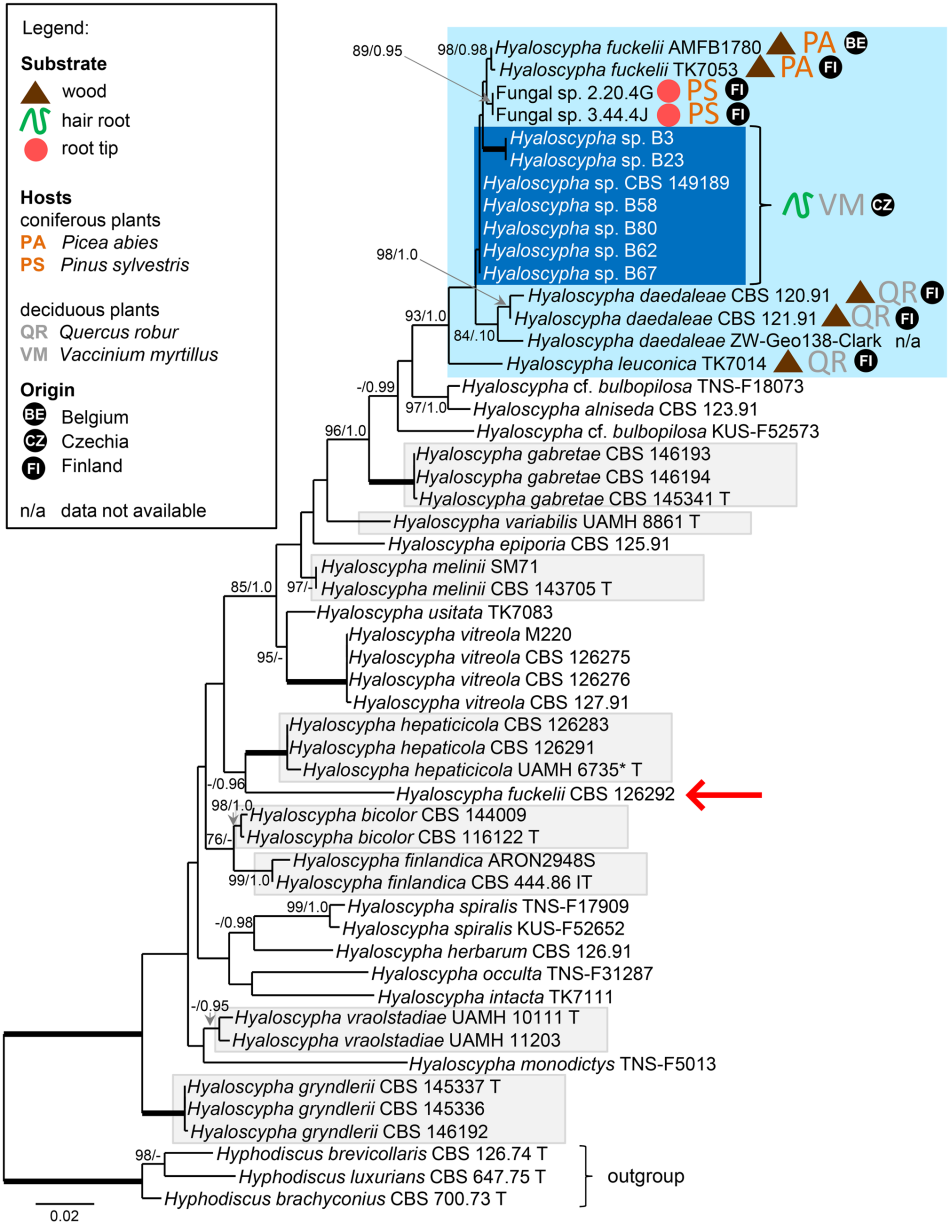
Maximum likelihood tree based on combined ITS and LSU rDNA sequences of members of *Hyaloscypha* s. str., showing the phylogenetic position of the new hyaloscyphoid isolates. Abbreviations T and IT after the name indicate ex-type and ex-isotype strains, respectively. Species names placed in grey boxes have been linked with mycorrhizal or root-endophytic lifestyle. Species names placed in the light blue box indicate closest relatives to our hyaloscyphoid isolates, which are placed in the blue box. The arrow points out the position of *Hyaloscypha fuckelii* CBS 126292, which is distant from *H. fuckelii* AMFB11780 and *H. fuckelii* TK7053 in the light blue box. Thickened branches indicate branch support with ML BS = 100% and PP values = 1.0. Branch support of nodes ≥ 75% ML and ≥ 0.95 PP is indicated above or below branches

Relationships of the new hyaloscyphoid isolates B3, B23, B58, B62, B67, B80, and CBS 149189 (see *Hyaloscypha* sp. above) obtained from hair root of *Vaccinium myrtillus* from Northern Bohemia were assessed in the phylogenetic analysis based on ITS-LSU sequences. Sequences of 43 strains representing 21 species of the genus *Hyaloscypha* s. str. available in GenBank were included in the analysis, as well as two unnamed fungal isolates that showed the highest conformity in the BLASTn searches. The ML and BI trees were highly concordant; the ML tree is shown in Fig. 4. The new hyaloscyphoid isolates clustered into a strongly supported clade consisting of *H. daedaleae, H. leuconica*, *H. fuckelii* and the two unnamed fungal isolates. The isolates B3 and B23 formed a monophyletic subclade while the rest of the isolates formed a separate lineage with little differentiation among them. These two groups differ slightly in their ITS sequences. The isolates B3 and B23 have identical ITS sequences and the isolates that clustered around the isolate B10 (CBS 149189) show 99.79–100% sequence identity, while ITS sequence identity between the two groups ranges from 98.75 to 98.95%. Based on this comparison and position in the phylogenetic tree, it is likely that they represent two different species. The closest named relatives to the two groups of the new hyaloscyphoid isolates are two strains of *H. fuckelii* (AMFB1780 and TK7053) and three strains of *H. daedaleae* (CBS 120.91, CBS 120.92, ZW-Geo138-Clark).

### First resynthesis

Compared to the non-inoculated control seedlings, those inoculated with *H.yaloscypha variabilis* B78, *Hyaloscypha* sp. CBS 149189 (B10) and *O. maius* B56 grew well, had green to green–brown leaves and did not show any symptoms of stress. In contrast, the control seedlings’ leaves were smaller and typically yellowish to reddish. Similar was true for the seedlings inoculated with *Mycena* sp. B14. The seedlings inoculated with *Gymnopilus* sp. B83 were overgrown by whitish mycelium and three of them died, hence they were excluded from the statistical analysis (see below). All pieces of the substrate collected for a verification of the inoculation success produced mycelium with ITS rDNA sequences matching those of the inoculated fungi, except the substrate inoculated with *Mycena* sp. B14 that produced no mycelium. The control substrate produced no mycelium.

**Fig. 5:**
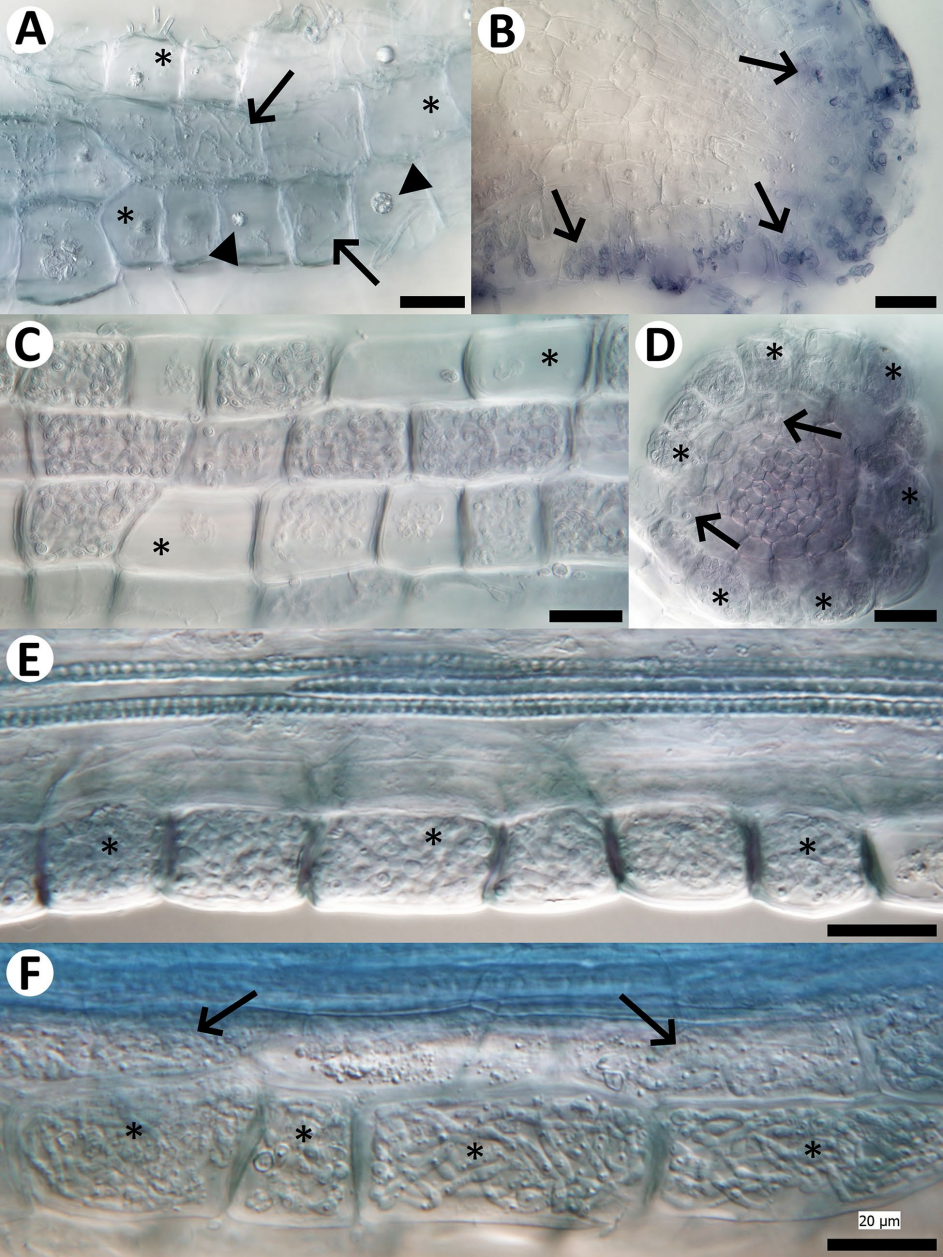
Results of the two resyntheses with *Vaccinium myrtillus* seedlings. **A:** Thin intracellular fungal hyphae resembling endophytic colonization rather than ericoid mycorrhiza (arrows point at some examples). Many rhizodermal cells are free of fungal colonization (asterisks mark some examples), the presence of intact nuclei (arrowheads point at some examples) suggests that the cells were metabolically active. First resynthesis, *Hyaloscypha variabilis* B78. **B:** Heavy fungal colonization at the tip of a hair root. Some cells are filled with fungal hyphae (arrows point at some examples) and this pattern resembles endophytic colonization rather than ericoid mycorrhiza. First resynthesis, *Gymnopilus* sp. B83. **C:** Typical ericoid mycorrhizal colonization by *Hyaloscypha* sp. B10 in contrast with non-colonized rhizodermal cells (asterisks mark some examples). Second resynthesis. **D:** Rhizodermal cells packed with dense hyphal coils typical for ericoid mycorrhiza (asterisks mark some examples). Note that while there are some signs of fungal colonization in the outer cortex (arrows), fungal hyphae never penetrate the stele/vascular tissues. Second resynthesis, *Hyaloscypha* sp. B10. **E:** Typical ericoid mycorrhizal colonization by *Hyaloscypha* sp. B10. Note that while nearly all rhizodermal cells are colonized (asterisks mark some examples), the cells below the rhizodermis do not show signs of fungal colonization. **F:** Another example of ericoid mycorrhizal colonization by *Hyaloscypha* sp. B10. Note that while all rhizodermal cells are colonized (asterisks), the fungal colonization extends also to the cells below the rhizodermis (arrows). All roots were cleared with 10% KOH, stained with trypan blue and observed using a compound microscope equipped with differential interference contrast as described in Materials and Methods. Scale bars = 20 μm

*Hyaloscypha variabilis* B78 and *O. maius* B56 colonized the roots of all inoculated seedlings but their colonization patterns did not resemble ericoid mycorrhiza (Fig. 5A). Similarly, the roots of the two surviving seedlings inoculated with *Gymnopilus* sp. B83 displayed locally abundant fungal colonization, including intracellular hyphae in the rhizodermis, but distinct from the typical ErM colonization pattern (Fig. 5B). Only *Hyaloscypha* sp. B10 CBS 149189 formed what morphologically corresponds to the ErM symbiosis (see below). The roots of the seedlings inoculated with *Mycena* sp. B14 were free of any fungal colonization, and therefore, this treatment was excluded from the statistical analysis. Roots of the non-inoculated control seedlings did not display any signs of fungal colonization.

### Second resynthesis

Only the seedlings inoculated with *Hyaloscypha* sp. B10 prospered better than the non-inoculated control and produced green to green–brown leaves. The substrate in all vessels inoculated with the basidiomycetes was colonized by whitish mycelia and in the vessels inoculated with *Gymnopilus* sp. B83, *Mycena* sp. JPK-117 and *Mycena* sp. JPK-152, they overgrew and subsequently killed 2–3 seedlings per treatment; consequently, these treatments were excluded from the statistical analysis. Dense intracellular hyphal coils typical for the ErM symbiosis were found only in the seedlings inoculated with *Hyaloscypha* sp. B10 (Fig. 5C–F). There were some extraradical hyphae visible on the surface of the roots of the seedlings inoculated with *Gymnopilus* JPK-155, *Mycena* sp. B14 and *Mycena* sp. JPK-151, but no intracellular hyphal colonization could be seen. In the non-inoculated control treatment, no mycelium was visible in the substrate and in and around the roots.

### Statistical analyses

In the first resynthesis, the inoculation significantly affected the seedlings' dry shoot weight (H = 9.789, p = 0.021) and the inoculation with *Hyaloscypha* sp. B10 significantly increased the seedlings' dry shoot weight in comparison with the non-inoculated control (16.62 ± 2.99 mg vs. 1.44 ± 2.99 mg, mean ± SE, p = 0.014). In the second resynthesis, the inoculation significantly affected the seedlings’ dry shoot weight (H = 12.730, *p* = 0.013) and fresh root weight (*N* = 16.634, *p* = 0.002). The inoculation with *Hyaloscypha* sp. B10 significantly increased the seedlings' dry shoot weight in comparison with the inoculation with *Gymnopilus* sp. JPK-155 (16.49 ± 1.30 mg vs. 1.30 ± 1.30 mg, *p* = 0.012) while its effect in comparison with the non-inoculated control (1.67 ± 1.30 mg) was marginally significant (*p* = 0.060). In comparison with the non-inoculated control, the inoculation with *Hyaloscypha* sp. B10 significantly increased the seedlings' fresh root weight (32.00 ± 3.22 mg vs. 1.2 ± 3.22 mg, *p* = 0.016).

## Discussion

Some basidiomycetous ErMF and saprobes like white rot fungi have a higher lignocellulolytic potential than the prominent ascomycetous ErMF *H. hepaticicola* (Bending and Read 1997; Vohník et al. 2012a), so the leading idea behind this study was that these would be more abundant in substrates containing higher amounts of lignin and cellulose, namely in decomposing wood. However, this was true only in the case of the samples from Mid-Norway where we had used an ascomycete-suppressing isolation medium. In contrast, the samples from Northern Bohemia subjected to a common medium without benomyl yielded a mycobiont spectrum dominated by helotialean ascomycetes, as is common for ericaceous plants dominating the archetypal peatland ecosystems of the Northern Hemisphere. While the resyntheses with saprobic basidiomycetes from Mid-Norway and Northern Bohemia brought at best inconclusive results, the Northern Bohemian samples yielded several hyaloscyphoid mycobionts with affinities to *H. fuckelii* sensu Kosonen et al. (2021) and *H. daedaleae* and their representative repeatedly formed what morphologically corresponds to the ErM symbiosis, and supporting the growth of the inoculated plants.

### Microscopic observations of natural colonization

Our investigations of root mycobionts of cultured and natural populations of *Vaccinium* spp. in Mid-Norway had started with microscopic observations of root colonization and resulted in the discovery of a new ErM morphotype that was named sheathed ericoid mycorrhiza. Due to the difficulties met during early attempts to isolate the respective mycobiont and later to amplify its rDNA using universal primers (Vohník et al. 2012a), microscopic observations remained the easiest way how to detect sheathed ericoid mycorrhiza and its hymenochaetoid mycobiont *Kurtia argillacea*. Hence, this study started with microscopic observations but contrary to our expectations, we did not find the characteristic basidiomycetous morphotype in *V. myrtillus* seedlings from Northern Bohemia, despite that their roots only occurred in decomposing wood. On the one hand, the surfaces of these hair roots were often covered by loose hyphal mantles. On the other hand, in comparison with *K. argillacea*, the hyphae forming these mantles were thinner and lacked clamp connections typical for many basidiomycetous mycobionts. Such a colonization pattern was not observed in the two resyntheses and the identity of the respective mycobiont(-s) thus remains unknown. The fact that these surface hyphae lacked clamp connections does not necessarily mean that they were of an ascomycetous origin. For example, some sebacinoid mycobionts can form ericoid mycorrhizae and cavendishioid ectendomycorrhizae, both comprising hyphae occurring on the root surface, and despite being basidiomycetes, they lack clamp connections (Setaro et al. 2006; Vohník et al. 2016). This question could be at least partially answered by transmission electron microscopy focused on the anatomy of the hyphal septa, which differ between asco- and basidiomycetes (e.g., Bonfante-Fasolo 1980; Selosse et al. 2007).

DSE are very common inhabitants of ericaceous roots (e.g., Massicotte et al. 2005; Vohník and Albrechtová 2011; Gorzelak et al. 2012) and while dark septate hyphae were common on the surface of the hair roots collected in Mid-Norway, they were absent in the hair roots from Northern Bohemia. Since only one isolate belonging to the *Phialocephala fortinii* s. l. – *Acephala applanata* species complex (Grünig et al. 2008) was recovered from these roots, it appears that moist decaying wood is not a suitable substrate for these mycobionts. A similar situation has been reported for ericaceous plants growing at an acidic wetland site in SW Canada (Hambleton and Currah 1997), suggesting that water content may be a primary edaphic factor influencing the distribution of DSE associating with core Ericaceae.

Ericoid mycorrhiza and DSE association are two morphologically distinct root-fungus symbioses characterized by fine intracellular hyphal coils in the rhizodermis of the hair roots of ericaceous plants and intracellular melanized microsclerotia, respectively. However, in some cases there seems to be a morphological continuum between these two colonization patterns (Vohník and Albrechtová 2011) and the samples from Mid-Norway provided one such example, namely thick melanized surface hyphae becoming thin, hyaline and coiled upon entering the host cell. This was sometimes connected with a formation of haustoria-like intracellular structures that seemed to be connected with intracellular hyphal coils occupying the same cells. Although these structures are occasionally observed in healthy-looking naturally colonized ericaceous hair roots (M. Vohník, personal observations), to our knowledge they have never been recorded in resynthesis studies and the identity of the respective mycobiont(-s) involved thus remains unknown.

### Detected fungal spectra

There was an apparent lack of *H. hepaticicola* in both sets of samples. While in the case of the samples from Mid-Norway this can be explained by the ascomycete-suppressing medium, the archetypal ErMF was probably absent and substituted by other mycobionts, including the ErMF *H. variabilis* and *O.idiodendron maius* and the new hyaloscyphoid fungi, in the case of the samples from Northern Bohemia. The new hyaloscyphoid fungi clustered with *H. fuckelii* sensu Kosonen et al. (2021) but not with *H. fuckelii* CBS 126292, suggesting that this species is in need of a taxonomic revision. In addition, they clustered with *H. daedaleae* and since these two taxa are known only as sexual saprobic morphs, it would be interesting to include them in a resynthesis experiment with an ericaceous host for comparison. In any case, previous studies have indicated a clear separation between asexual root-symbiotic and sexual saprobic *Hyaloscypha* s. str. spp. (Fehrer et al. 2019; Vohník et al. 2022) and our study seems to be the first case where representatives of the different reproductive and trophic morphs intermingle in one statistically supported phylogenetic clade. Additional members of the clade are two isolates from root tips of *Pinus sylvestris* (Fungal spp. 2.20.4G/GenBank KM068412 and 3.44.4 J/KJ649999) and except decaying wood, the clade is thus known from both conifer (*Picea abies* and *P. sylvestris*) and deciduous (*Quercus robur* and *V. myrtillus*) hosts from Belgium, Czechia, and Finland. Such a relatively broad host and distribution range is reminiscent of other asexual root symbionts in the genus *Hyaloscypha* s. str., namely *H. variabilis* (Hambleton and Sigler 2005; Vohník et al. 2013) and *H. gryndleri* (Vohník et al. 2022; Daghino et al. 2022).

The genus *Pezicula* (asexual morph *Cryptosporiopsis*) belongs to Dermateaceae (Helotiales) and contains more than 130 species (MycoBank, mycobank.org, accessed 11/8/2022) that often produce apothecial ascomata on the bark of temperate woody plants (Verkley 1999). *Pezicula* comprises both saprobes and symbionts (pathogens and endophytes, possibly also mutualists) of a wide range of hosts (Sieber 2007; Chen et al. 2016) and many species are potent producers of biologically active secondary metabolites (e.g., Stillwell et al. 1969; Fisher et al. 1984; Noble et al. 1991; Schulz et al. 1995). Isolates belonging to this genus are from time to time obtained from ericaceous roots (e.g., Verkley et al. 2003; Sigler et al. 2005; Zijlstra et al. 2005; Walker et al. 2011) and the most common species include *P. brunnea*, *P. ericae*, *P. radicicola,* and *P. rhizophila*, the first morphologically described *Pezicula* (*Cryptosporiopsis*) species from ericaceous roots (Verkley et al. 2003). However, their symbiotic status is not clear and may range from (weak) pathogenicity to mutualism. For example, due to its repeated isolations from surface-sterilized healthy roots of several ericaceous hosts, Verkley et al. (2003) regarded *P. rhizophila* as an endophytic fungus. Zijlstra et al. (2005) reported that *Calluna vulgaris* seedlings inoculated in vitro with *P. rhizophila* CBS 109839 showed increased nitrogen content compared to the non-inoculated control seedlings, but no information about root colonization was provided. Finally, Walker et al. (2011) reported that in their sterile resynthesis system, two *P. ericae* isolates formed “hyphal complexes typical for ericoid mycorrhiza” in the roots of *Vaccinium uliginosum*, thus demonstrating a “potential to establish ErM associations”, despite that the colonization of the roots “tended to be low” and no photo-documentation was provided. In this study, the OTU NB-03 from Northern Bohemia most likely represented *P. neosporulosa* and the number of its isolates equaled that of the new hyaloscyphoid fungi. *Pezicula neosporulosa* was described as an endophyte/parasite of *Abies* spp. from China and the Netherlands (Yuan and Verkley 2015) and to our knowledge, nothing is known about its functioning in ericaceous roots.

The mycobiota of the ericaceous hair roots regularly comprises a basidiomycetous component that is mainly formed by the difficult-to-cultivate sebacinoid ErMF, but also includes non-sebacinoid ErMF, various endophytes and pathogens and some typical EcM and saprobic fungi (see Vohník (2020) and references therein). While the Mid-Norwegian samples yielded one serendipitoid isolate, shown in another study to form what morphologically corresponds to the ErM symbiosis (Vohník et al. 2016), no sebacinoid fungi were isolated from the Northern Bohemian samples. Contrary to the results of, e.g., Bougoure et al. (2007) and Lorberau et al. (2017), and despite the fact that the sampling sites were in the middle of two coniferous forests, we did not obtain any EcM fungi. However, both sets of samples yielded several basidiomycetous OTU that could be linked to genera traditionally reserved for saprobes, namely *Coprinellus*, *Gymnopilus*, *Hypochnicium* and *Mycena*, the last being the most prevalent. *Mycena* (Mycenaceae, Agaricales) contains more than 1900 species (mycobank.org, accessed 23/8/2022) and while these are typically saprobic, they also associate with plant roots (see Harder et al. (2021) and references therein) and engage in the orchid mycorrhizal symbiosis (e.g., Ogura-Tsujita et al. 2009; Zhang et al. 2012; Lee et al. 2015). *Mycena* are not uncommon in ericaceous roots (see Grelet et al. (2017) and references therein) but the mode of their interactions needs to be clarified (also see below). It is well known that many fungal pathogens may have latent endophytic stages that become harmful when the host is weakened (Schulz and Boyle 2006) and that saprobes may colonize dying or already dead cells of a hair root that otherwise looks healthy (Grunewaldt-Stöcker and von Alten 2016), possibly explaining our observations of saprobic basidiomycetes in *Vaccinium* spp. hair roots.

### Resyntheses

Our at best inconclusive results in the resyntheses with saprobic basidiomycetes do not support previous observations of their beneficial effects on ericaceous hosts (Grelet et al. 2017). However, under certain scenarios, they can be beneficial for the growth of ericaceous plants even without forming a root-fungus symbiosis (Vohník 2020). For example, under natural conditions they never interact with fungus-free roots and there is an indication that they might benefit ericaceous plants through interactions with ErMF (Vohník et al. 2012b). More experimental work is apparently needed to resolve this issue, ideally employing a combined inoculum containing both ErMF and asymbiotic saprobic fungi.

A representative of the new hyaloscyphoid clade repeatedly formed intracellular hyphal structures identical to those formed by typical ErMF and since it also supported the growth of the inoculated blueberry seedlings, there is a good chance that it represents a new ErMF, similarly to the recently described *H. gryndleri* (Vohník et al. 2022). However, this must be confirmed by more experimental studies, ideally employing other members of the clade and perhaps also isolates of the sexual saprobic *H. daedaleae* and *H. fuckelii* for comparison.

## Conclusions

Rather than providing an exhaustive account of fungi inhabiting the roots of ericaceous plants growing on decomposing wood, this study offers a complex peek beyond the traditional scheme “ericoid mycorrhiza = acidic peaty substrates”, revealing once again how little we know about this important root-fungus association. Our observations do not support the view that typical EcM and saprobic basidiomycetes are mycorrhizal symbionts of ericaceous plants, but this issue is far from being solved and more experimental work is needed. Mountainous forested areas in Central Europe seem to be an unexpectedly rich reservoir of new root-symbiotic hyaloscyphoid fungi (Fehrer et al. 2019; Vohník et al. 2022, this study) and we encourage their research especially in the hitherto overlooked non-peat substrates. Such substrates are also found in the Southern Hemisphere (especially Australia and South Africa) where many ericaceous plants thrive in sandy soils where wood and sclerophyllous leaves represent major (often the only available) sources of nitrogen and phosphorus.

## Data Availability

The sequences obtained in this study were deposited in GenBank at NCBI, herbarium specimens (as dried cultures) were deposited in the Herbarium of the Institute of Botany, Czech Academy of Sciences, Průhonice, Czechia (PRA), and living cultures were deposited in the collection of the Westerdijk Fungal Biodiversity Institute in Utrecht, the Netherlands (CBS).
